# Non-alcoholic fatty liver disease in obese subjects as related to increasing insulin resistance and deteriorating glucose control: Three years of follow-up from a longitudinal survey

**DOI:** 10.1007/s40200-023-01378-z

**Published:** 2023-12-30

**Authors:** Thomas Forst, Isabel Botz, Matthias Berse, Stephan Voswinkel, Mares-Elaine Strempler, Sybille Baumann, Maria Marinez

**Affiliations:** 1 CRS Clinical Research Services Mannheim GmbH, Grenadierstrasse 1, 68167 Mannheim, Germany; 2 CRS Clinical Research Services Berlin GmbH, Berlin, Germany; 3 MLM Laboratories Mönchengladbach, Mönchengladbach, Germany

**Keywords:** Obesity, Insulin resistance, Diabetes mellitus type 2, Non-alcoholic fatty liver disease (NAFLD), Adipocytokines

## Abstract

**Purpose:**

This observational trial was performed to evaluate liver parameters in overweight or obese subjects in the context of insulin resistance and glucose control over time.

**Subjects/Methods:**

Insulin resistance, glucose control and several parameters for liver integrity were monitored in 177 overweight (BMI > 28 kg/m2) subjects over a mean of 30 months. Volunteers were categorized according to insulin resistance (HOMA_IR_ score) and glucose control in subjects with normal glucose control (NGT), impaired glucose control (IGT), or diabetes mellitus type 2 (T2DM). Liver fat and fibrosis were evaluated by sonographic elastography (FibroScan®) and clinical scores, such as the AST/ALT ratio, fatty liver index (FLI), and NAFLD fibrosis score (NFS).

**Results:**

Liver fat fraction as estimated by the controlled attenuation parameter (CAP), and the FLI were significantly higher in subjects with T2DM compared to IGT and NGT. While fasting insulin levels and the HOMA_IR_ score continuously increased over time, no change in CAP or FLI occurred during follow up. CAP was correlated with FLI (*r* = 0.50; *p* < 0.0001) and the HOMA_IR_ score (*r* = 0.32; *p* < 0.0001). An inverse correlation was observed between serum adiponectin levels and FLI (*r* = -0.37; *p* < 0.0001), the HOMA_IR_ score (*r* = -0.19; *p* < 0.001, and CAP (*r* = -0.15; *p* < 0.01).

**Conclusions:**

In subjects with a BMI ≥ 28 kg/m^2^, liver fat fraction is significantly elevated in those with T2DM compared to IGT or NGT. Liver fat fraction is associated with deteriorating insulin sensitivity and loss of glucose control. Despite a continuous increase in insulin resistance, no change in liver fat content or stiffness occurred over 30 months.

## Introduction

Obesity has emerged as one of the most prominent metabolic disorders with serious consequences for health care systems worldwide. Obesity is most often accompanied by several health-threatening conditions, such as fatty liver disease, insulin resistance, systemic inflammation, lipid disorders and deteriorating glucose control. An increase in adipose tissue and the development of insulin resistance with a high flow of free fatty acids from adipocytes to the liver is a central mechanism in the development of fatty liver disease and liver fibrosis [1, 2]. Since treatment and prognosis of infective hepatitis have markedly improved in recent years, NAFLD has become the most common cause of severe liver injury, with a high risk of progressing to steatohepatitis, liver cirrhosis, or hepatocellular carcinoma [3, 4]. Beyond these hepatic complications, NAFLD markedly increases the risk of cardiovascular complications, heart failure, and chronic kidney disease [5–9]. Insulin resistance is hypothesized to be the main pathophysiological link between NAFLD and these non-hepatic complications [10]. Currently, medical societies realize that NAFLD is a significant health care challenge with a high need for early medical attention.

The results presented here are generated from a longitudinal observational trial tracking several biomarkers and clinical and sonographic parameters for insulin resistance and glucose control over a period of 30 months in overweight or obese subjects. The study design, methods and baseline data of the study participants have been published in detail previously [11].

## Materials and methods

### Patients and study design

The data presented here are retrieved from two follow-up visits of a longitudinal survey in overweight or obese subjects. The study was performed in accordance with the Declaration of Helsinki. The study was approved by the local ethical review board and was registered in the German registry of clinical studies (registration number DRKS00017516).

Criteria for inclusion in the study were a BMI greater than 28 kg/m^2^, male or female subjects between 18 and 80 years, subjects to be able to understand and follow the study instructions, and a signed informed consent. Subjects were excluded if they had diabetes mellitus type 1, maltose malabsorption, acute gastrointestinal disorders, systemic treatment with corticoids, pregnant or nursing women, active implantable medical devices (e.g., pacemaker), ascites, acute hepatitis, positive results in hepatitis or human immunodeficiency test, or a history of alcohol abuse.

Subjects were categorized as patients with diabetes mellitus type (T2DM) according to their medical history based on increased fasting glucose levels, HbA1c levels, or an oral glucose test. Patients with T2DM were included in the study independent from their glucose lowering treatments (lifestyle, metformin, sulfonylurea, DPP-IV inhibitors, sGLT-2 inhibitors, GLP-1 receptor agonists, or insulin. Participants without a history of diabetes mellitus type 2 (T2DM) underwent a standardized oral glucose tolerance test containing 75 g glucose solved in 300 ml water at all three visits. Subjects were allocated to the IGT group if their blood glucose concentration at 120 min after glucose intake was between 140 and 199 mg/dL (7.8 and 11.0 mmol/L). Subjects with a glucose level greater than 199 mg/dL two hours after glucose intake were allocated to the T2DM group.

At the baseline visit (V1) and at two follow-up visits after approximately 15 (V2) and 30 months (V3), the study participants entered the study sites in the morning after fasting overnight for at least 10 h. Fasting blood samples were taken for the measurement of glucose, insulin, aspartate aminotransferase (AST), alanine aminotransferase (ALT), gamma glutamyl transpeptidase (gamma-GT), triglycerides, thrombocytes, serum albumin, leptin and adiponectin levels.

Insulin, AST, ALT, gamma-GT, triglycerides, and albumin were measured in serum. Fasting glucose was measured in sodium fluoride (NaF) plasma using Cobas® 6000 analysers (Roche, Germany). Thrombocytes were analysed in EDTA blood using a Sysmex XN-1000 blood counter (Sysmex Deutschland GmbH, Germany). Leptin was analysed in serum with an ELISA from DRG Instruments. Total adiponectin was analysed in serum with an ELISA from R&D Systems.

### Clinical and biomarker scores

#### HOMA_IR_ score

The HOMA_IR_ score was used to estimate insulin resistance [12]. The HOMA_IR_ score was calculated from fasting glucose and insulin concentrations as follows:

$${\mathrm{HOMA}}_{\mathrm{IR}}=\mathrm{fasting}\;\mathrm{insulin}\;\lbrack\mathrm\mu\mathrm U/\mathrm{mL}\rbrack\times\mathrm{fasting}\;\mathrm{glucose}\;\lbrack\mathrm{mg}/\mathrm{dL}\rbrack\;\mathrm{divided}\;\mathrm{by}\;405.$$

#### Fatty liver index (FLI)

FLI is an established measure for a non-invasive estimate of liver fat content [13]. The FLI value is calculated based on the laboratory markers triglycerides and gamma-GT and the body composition markers BMI and waist circumference according to the formula:

$$\mathrm{FLI}=\mathrm e^{\mathrm y}\;\mathrm{divided}\;\mathrm{by}\;(1+\mathrm e^{\mathrm y})\times100,$$

where e is Euler’s number 2.71828 and y is 0.953 × ln (triglycerides, mg/dL) + 0.139 × BMI, kg/m^2^ + 0.718 × ln (gamma-GT, U/L) + 0.053 × waist circumference, cm – 15.745) [14].

#### NAFLD fibrosis score (NFS)

NFS is a validated score for estimating the severity of liver fibrosis.

This composite score is calculated based on the factors age, hyperglycaemia (i.e., impaired fasting glucose [IFG]/diabetes), BMI, platelet count (thrombocytes), AST/ALT ratio (AAR), and albumin as follows:

$$\mathrm{NFS}=-1.675+(0.037\times\mathrm{Age}\;\lbrack\mathrm{years}\rbrack)+(0.094\times\mathrm{BMI}\;\lbrack\mathrm{kg}/\mathrm m^2\rbrack)+(1.13\times\mathrm{IFG}/\mathrm{diabetes}\;\lbrack\mathrm{yes}=1,\;\mathrm{no}=0\rbrack)+(0.99\times\mathrm{AAR})-(0.013\times\mathrm{thrombocytes}\;\lbrack\times10^9/\mathrm L\rbrack)-(0.66\times\mathrm{albumin}\;\lbrack\mathrm g/\mathrm{dL}\rbrack).$$

#### Liver fat and stiffness

Liver fat and stiffness were measured using transient elastography (FibroScan®, Echosens, Paris, France). The FibroScan® device evaluates liver fat content given by the controlled attenuation parameter (CAP), expressed as decibels per metre (dB/m), as well as liver stiffness given as the Young’s modulus (E), expressed as kilopascal (kPA) [15, 16].

#### Statistical analysis

For statistical comparisons between study groups and other variables of interest (e.g., HOMA_IR_ and fibrosis categories), the type of analysis was chosen based on the type of data (numerical/categorical) and number of groups compared.

If a numerical variable was compared among more than two groups, 95% confidence intervals of the numerical variables for each group were added to the summary statistics. The overall influence of the grouping variable was assessed by the p value of the F test of a simple analysis of variance (ANOVA) model using the grouping variable as the single independent variable. Pairwise differences were assessed by calculating the least square mean difference and the 95% confidence interval. The statistical significance of differences was assessed using the p value of the corresponding t test.

If a numerical variable was compared between two groups, 95% confidence intervals of the numerical variables by group were added to the summary statistics. Groups were compared by calculating the arithmetic mean difference, parametric 95% confidence interval, and p value of the corresponding t test.

If a binary variable was compared among two or more groups, groups were compared overall and pairwise using a chi-square test.

Furthermore, linear regressions were performed using liver stiffness and liver fat as dependent variables and HOMA_IR_, AST/ALT ratio, FLI and NFS as independent variables. Pearson correlation coefficients were calculated for all variables with a p value < 0.05 in the regression analyses.

For group comparisons, raw p values are given. A p value < 0.05 was considered statistically significant.

## Results

At baseline (V1), 301 overweight subjects with a BMI > 28 kg/m2 were enrolled in the study and were categorized according to their insulin sensitivity and diabetic status [11]. After a mean of 15 months (V2) 240, and after a mean of 30 months (V3) 177 subjects were available for follow up. The data presented here rely on the subset of 177 subjects who could be followed for the whole duration of 871 ± 142 days (mean ± SD). Of these 177 subjects (72 female, 105 male), 88 were with NGT, 26 were with IGT, and 63 subjects appeared with T2DM. Over the entire observational period, 18 out of 88 subjects (20%) deteriorated from NGT to IGT, and 2 out of 26 (8%) deteriorated from IGT to T2DM.

**Fig. 1:**
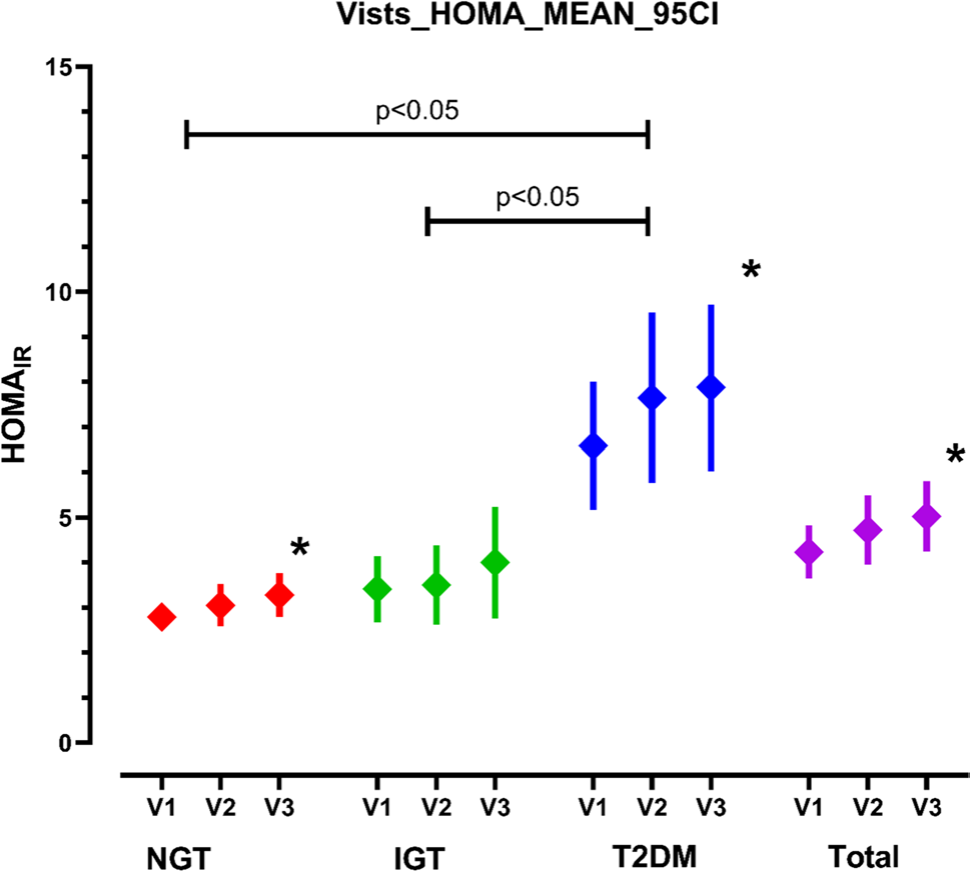
HOMA_IR_ score at baseline (V1) and after a mean of 15 (V2) and after a mean of 30 months (V3) in overweight or obese subjects with normal glucose tolerance (NGT), impaired glucose tolerance (IGT) or type 2 diabetes mellitus (T2DM) (mean (95% CI); * *P* < 0.05 vs. baseline)

As shown in Table 1, the mean body weight over time slightly increased in the NGT group, remained unchanged in the IGT group, and slightly declined in the T2DM group. BMI increased by 0.49 ± 1.9 kg/m2 (mean ± SD; *p* < 0.05) in the NGT group and declined by 0.6 ± 1.8 kg/m2 (*p* < 0.05) in the T2DM group. Overall fasting insulin levels increased by 1.7 ± 9.9 mU/L at V2 and by 2.33 ± 8.6 mU/l at V3 (*p* < 0.001). This was mainly driven by an increase in insulin levels in the NGT group by 1.9 ± 5.7 (*p* < 0.01) and by 2.9 ± 11.9 mU/L (p = 0.06) in the T2DM group. Insulin resistance as evaluated with the HOMA_IR_ score was significantly higher in the T2DM group than in the IGT and NGT groups (Table 1). In all subjects, the HOMA_IR_ score increased by 0.5 ± 3.1 (*p* < 0.05) after 15 months and by 0.8 ± 2.9 (*p* < 0.001) after 30 months (Fig. 1). The increase in insulin resistance in the overall group was mainly driven by an increase in HOMA_IR_ of 0.49 ± 1.5 (*p* < 0.01) in the NGT group, and by 1.3 ± 4.3 (*p* < 0.05) in the T2DM group.

**Table 1:** Clinical parameters at visits 1, 2 and 3 (mean (95% CI); * *p* < 0.05 vs. baseline; § *p* < 0.05 vs. NGT; $ *p* < 0.05 vs. IGT at V3)

	NGT			IGT			T2DM			Total		
	V1	V2	V3	V1	V2	V3	V1	V2	V3	V1	V2	V3
Time of follow-up (days; mean ± SD)	Baseline	488 ± 131	885 ± 155	Baseline	486 ± 125	883 ± 140	Baseline	456 ± 116	846 ± 121	Baseline	477 ± 125	871 ± 142
Body weight (kg; mean (95% CI))	96,3 (93,7–98.9	97.1 (94.2–100.0)	97,8 * (94.8–100.8)	91,0 (84.8–97.1	90.5 (84.8–97.1)	91.5 (85.3–97.7)	98.9 (95.6–102.2)	98,4 (94.7–102.0)	97,1 *,$ (93.9–110.2)	95.7 (94.5-98.4)	95.3 (94.4–98.6)	98.7 (94.5)98.7)
BMI (kg/m²; mean (95% CI)	31.5 (31.0–31.9)	31,8 (31.1–32.4)	31,9 * (31.3–32.6)	32,2 (30.8–33.7)	32,1 (30.6–33.7)	32,5 (30.8–34.1)	33,5 (32.6–34.4)	33,3 (32.2–34.4)	32,9 *, § (32.0–33.9)	32,3 (31.8–32.8)	32,4 (31.8–32.9)	32,4 (31.8–32.9)
Insulin (mU/L; mean (95% CI))	11.3 (10.1–12.5)	12,3 (10.4–14.1)	13,2 * (11.4–15.0)	13,2 (10.7–15.8)	13,9 (10.7–17.1)	15,8 (11.4–20.2)	17,1 (13.4–20.8)	20,6 (13.8–27.4)	20,0 *, §,$ (14.1–25.9)	13,7 (12.1–15.2)	15,4 * (12.8–18.0)	16,0 * (13.6–18.4)
HOMA_IR_; (mean (95% CI))	2,8 (2.5–3.1)	3,1 (2.6–3.1)	3,3* (2.8–3.8)	3,4 (2.7–4.2)	3,5 (2.6–4.4)	4,0 (2.8–5.2)	6,6 (5.2–8.0)	7.7 (5.8–9.5)	7,9 *, §,$ (6.0–9.7)	4,2 (3.6–4.8)	4,7* (4.0–5.5)	5,0 * (4.3–5.8)
AST/ALT Ratio (mean (95% CI)	0.96 (0.89–1.02)	1.0 (0.95–1.08)	0,98 (0.91–1.05)	0,91 (0.80–1.02)	0,91 (0.80–1.00)	0,94 (0.82–1.06)	0,86 (0.78–0.94)	0,89 (0.82–0.97)	0,87 § (0.80–0.95)	0,92 (0.87–0.96)	0,96 (0.91–1.00)	0,94 (0.89–0.98)
FLI (mean (95% CI)	69,6 (65.8–73.4)	71,1 (66.7–75.5)	72,0 (67.8–76.3)	72,0 (61.1-80.3)	71,8 (62.1–80.3)	71,4 (79.4–87.9)	85,0 (81.7–88.2)	85,2 (81.1–89.2)	83,6 §,$ (79.4–87.9)	75,2 (72.5–78.0)	76,1 (73.0–79.3)	76,1 (73.0–79.1)
NFS (mean (95% CI)	-2,1 (-2.3 - -1.9)	-2,4 (-3.2 - -1.6)	-2,0 (-2.2 - -1.8)	-1,8 (-2.3 - -1.4)	-1,8 (-2.2 - -1.3)	-1,8 (-2.2 - -1.4)	-0,3 (-0.6 - -0.04)	-0,34 (-0.6 - -0.05)	-0,5 §,$ (-0.8 - -0.2)	-1,4 (-1.6 - -1.2)	-1,6 (-2.0 - -1.2)	-1,5 (-1.6 - -1.3)
CAP (dB/m; mean (95% CI))	278 (266–290)	271 (257–284)	278 (264–291)	273 (246–301)	290 (262–318)	280 (259–301)	314 (297–332)	318 (304–333)	314 §,$ (298–331)	290 (281–300)	290 (281–300)	291 (282–301)
E (kPA; (mean (95% CI))	6,9 (4.2–9.5)	6,0 (4.3–7.7)	5.6 (3.9–7.2)	5,4 (3.9–6.9)	6,0 (4.0–8.1)	5,2 (4.4–6.0)	7,7 (6.4–9.0)	11,3 (7.3–15.3)	7,5 (6.3-8.6)	6,9 (5.5–8.3)	7,9 (6.2–9.6)	6,2 (5.3–7.1)

**Fig. 2:**
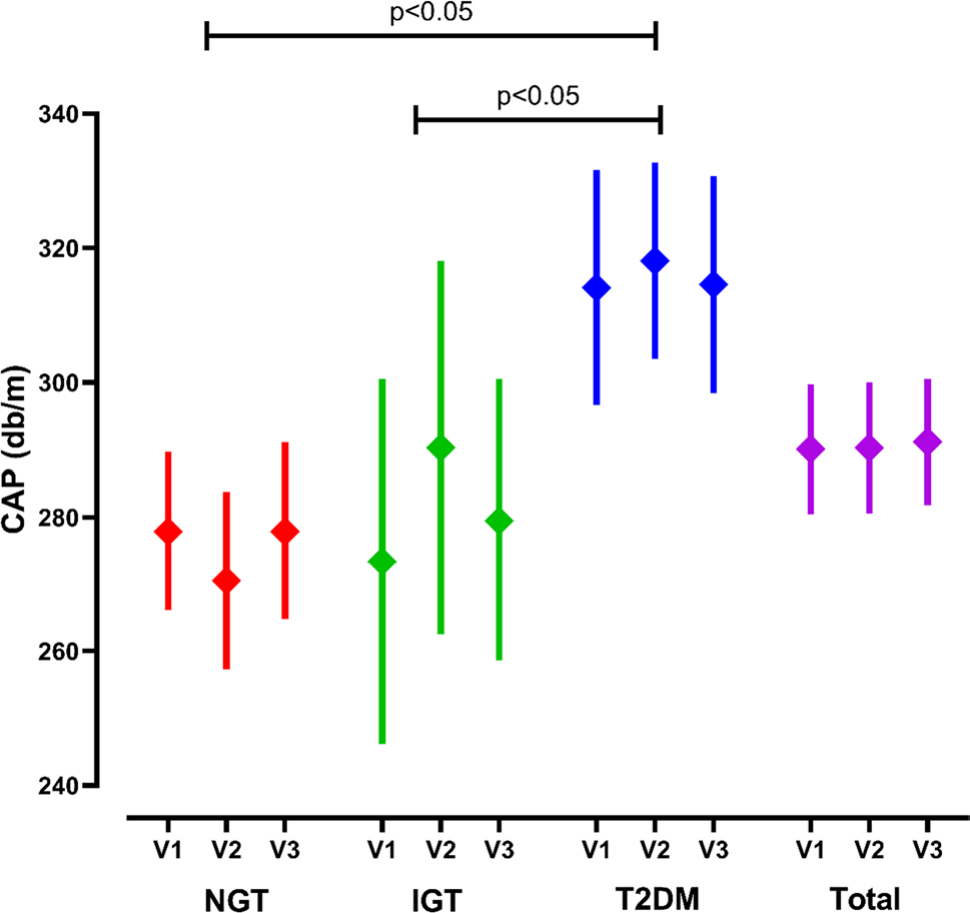
Controlled attenuation parameter (CAP) after after a mean of15 (V2) and after a mean of 30 months (V3) in overweight or obese subjects with normal glucose tolerance (NGT), impaired glucose tolerance (IGT) or type 2 diabetes mellitus (T2DM) (mean (95% CI); * *P* < 0.05 vs. baseline)

Measurement of liver fat content (CAP) as recorded with sonographic elastography revealed a significantly higher liver fat fraction in overweight subjects with T2DM compared to overweight subjects with NGT or IGT (Fig. 2). When the subjects were divided categorically into those with IR (HOMA_IR_ ≥ 2) or without IR (HOMA_IR_ < 2), those with IR exhibited a significantly higher CAP than insulin-sensitive overweight subjects (297 (286–308) dB/m vs. 270 (241–280) db/m: mean (mean (95% CI); *p* < 0.001). Linear regression analysis revealed a linear relationship between the HOMA_IR_ score and the CAP measurement (r = 0.32; *p* < 0.0001). No significant change in CAP could be observed over time.

In accordance with the CAP measurements, FLI, as a clinical score for liver fat content, was significantly higher in overweight subjects with T2DM than in the IGT and NGT groups (Table 1). FLI was significantly higher in those patients with HOMA_IR_ ≥ 2 compared to those with a HOMA_IR_ < 2 (78.6 (75.4–81.8) vs. 67.1 (60.0–74.3); *p* < 0.0001). The liver fat fraction evaluated by CAP showed a close correlation with FLI (r = 0.50; *p* < 0.0001).

Due to a change in the study design after the baseline investigations, leptin and adiponectin concentrations were only measured at V2 and V3. Cross-sectional group comparisons for leptin, adiponectin and leptin adiponectin ratio (LAR), without looking into changes over time, were performed. As shown in Table 2, serum leptin concentrations were not significantly different between the three groups of overweight subjects neither at baseline nor at follow-up visits. In accordance with liver fat content and insulin resistance, adiponectin levels were reduced in overweight subjects with T2DM compared to those with NGT or IGT at all visits during the trial. No significant difference could be observed between the subjects with NGT or IGT. When the subjects were categorized according to their CAP measurements in three categories with a liver fat fraction less than 5%, 5 to 33%, or more than 33%, the group of overweight subjects with a liver fat fraction above 33% were found to have significantly higher HOMA_IR_ scores and significantly lower adiponectin levels compared to those groups with a fat fraction of less than 33% or less than 5% (Table 3). An inverse correlation was found between CAP and adiponectin levels (r = -0.15, *p* < 0.01) and between FLI and adiponectin levels (r = -0.38; *p* < 0.0001). A linear correlation was observed between CAP and HOMA_IR_ (r = 0.32; *p* < 0.0001).

**Table 2:** Adiponectin and leptin serum levels and the leptin/adiponectin ratio at visit 2 (mean (95% CI); § *p* < 0.05 vs. NGT; $ *p* < 0.05 vs. IGT)

	NGT		IGT		T2DM		Total	
	V2	V3	V2	V3	V2	V3	V2	V3
Adiponectin (µg/mL)	6.5 (5.7–7.3)	6.8 (6.0–7.7)	7.6 (5.7–6.4)	7.9 (5.9–10.0)	5.3 §,$ (4.4–6.3)	5.5 §,$ (4.8–6.2)	6.3 (5.7–6.9)	6.5 (5.9–7.1)
Leptin (ng/mL)	9.6 (7.5–11.8)	9.1 (7.4–10.9)	12.5 (8.3–16.8)	11.7 (8.5–14.9)	12.0 (9.5–14.6)	11.5 (9.1–13.8)	10.9 (9.4–12.4)	10.3 (9.1–11.6)
Leptin/adiponectin ratio	1.7 (1.4–2.1)	1.6 (1.25–1.85)	1.9 (1.3–1.9)	2.0 (1.3–2.7)	2.7 § (2.1–3.3)	2.4 § (1.9–2.9)	2.1 (1.8–2.4)	1.9 (1.7–2.2)

**Table 3:** HOMA_IR_ score and adiponectin levels according to liver fat fraction (CAP) in overweight and obese subjects (mean (95% CI); § *p* < 0.05 vs. CAP < 5%; $ *p* < 0.05 vs. CAP 5–333%)

	CAP fat fraction < 5%	CAP fat fraction 5–33%	CAP fat fraction > 33%
HOMA_IR_	2.66 (2.14–3.18)	3.82 (2.50–5.14)	6.03 (4.92–7.13) §,$
Adiponectin (µg/ml)	7.43 (5.90–8.96)	8.03 (6.41–9.66)	5.82 (5.15–6.48) §,$

While NFS was significantly higher in the overweight subjects with T2DM than in the nondiabetic subjects, no significant difference between the groups could be observed regarding liver stiffness as measured with elastography (E) (Table 1). Neither NFS nor E changed over time in the three overweight or obese groups.

## Discussion

According to a recent meta-analysis, it is estimated that approximately one-third of the overall population worldwide is affected by an increase in liver fat content [17]. NAFLD has emerged as the most frequent liver disease, with an estimated prevalence of approximately 25% in the overall population and 70%-80% in high-risk groups, such as obese subjects or patients with T2DM [18–20].

Our data obtained from 177 overweight subjects (BMI ≥ 28 kg/m2) are consistent with published data, showing an incidence of NAFLD in approximately 80% of overweight subjects with a BMI ≥ 28 kg/m2 [11]. Insulin resistance and the liver fat fraction were found to be significantly higher in overweight or obese subjects with T2DM than in those with NGT or IGT. No significant difference in insulin resistance or liver fat fraction was observed between obese subjects with NGT or IGT. While insulin resistance continuously increased over 30 months of follow up, liver fat fraction as estimated by CAP or FLI remained almost unchanged. It remains to be established whether this is an indicator of a varying progression rates of insulin resistance and liver fat content over time. NFS and sonographic liver elastography indicated increased liver stiffness and fibrosis in obese subjects with T2DM compared to those without T2DM. No significant change in these parameters could be observed over the period of 30 months.

Insulin resistance is considered to represent a cornerstone pathway in the development of NAFLD and associated hepatic and extrahepatic complications [20, 21]. Our results confirm previous data from Motamed et al. that even in subjects without T2DM, insulin resistance was found to be a strong predictor of NAFLD [22]. In our study, liver fat content was shown to be associated with IR, as indicated by HOMA_IR_ categories. Moreover, regression analysis revealed a strong association between HOMA_IR_, CAP and the FLI.

Leptin and adiponectin are important adipocytokines that are almost exclusively released from visceral adipocytes. Decreased adiponectin levels and an increased LAR are considered indicators of adipocyte dysfunction and a shift to proinflammatory and profibrotic remodelling of the adipose tissue [23, 24].

In addition to the well-documented low adiponectin levels in overweight subjects [25, 26], our data show even lower adiponectin levels in overweight subjects with T2DM compared to overweight subjects without T2DM. Adiponectin mediates insulin-sensitizing effects by binding to its receptors AdipoR1 and AdipoR2, leading to activation of adenosine monophosphate-dependent kinase (AMPK), PPAR-α, and presumably other yet-unknown signalling pathways [27]. Consistent with these adiponectin signalling pathways, our data reveal an inverse association among adiponectin levels, liver fat content (CAP; FLI) and insulin resistance as expressed by HOMA_IR_. No association was observed between adiponectin levels and the indicators of liver fibrosis (E; NFS).

No difference in leptin levels were found between the overweight subjects with or without T2DM. This finding is consistent with a publication by Thorand et al., showing a stronger association between adiponectin levels and T2DM compared to leptin levels and T2DM in obese subjects [28].

LAR has been proposed as an even stronger indicator of insulin resistance and T2DM compared to the level of each single adipocytokine alone [24, 29, 30]. In our study, LAR did not provide any additional information on insulin resistance, or the liver fat content compared to single adiponectin levels alone.

The strength of the study is to document the time course of liver fat and liver stiffness in accordance to insulin resistance and glucose control in obese subjects with and without T2DM. Liver fat fraction was found to be elevated in those subjects with higher insulin resistance and with impaired glucose control. Despite a continuous increase in insulin resistance found over a mean of 30 months, no significant change in liver fat content or stiffness could be observed during that time.

There are some important limitations that need to be considered. It might be conceivable that, despite a deterioration of insulin sensitivity, the observational period of 30 months was too short to identify a significant deterioration in liver fat fraction or stiffness. Future studies with longer observational periods should address whether liver fat and stiffness might increase in accordance with an increase in insulin resistance on longer time scales.

The IGT group with 26 subjects was relatively small compared to the NGT group with 88 subjects and the T2DM group with 63 subjects. Therefore, the results obtained for the IGT subgroup should be interpreted with caution.

Leptin and adiponectin levels were only measured at V2 and V3. Therefore, only cross-sectional analysis and no evaluation of changes over time were performed.

## Conclusions

In overweight or obese subjects with a BMI ≥ 28 kg/m^2^, T2DM is associated with increased insulin resistance, increased liver fat and stiffness, and reduced adiponectin levels compared to non-diabetic subjects independent from NGT or IGT. Although insulin sensitivity steadily declined over time in all observed overweight subjects, no significant change in liver fat or liver stiffness was observed over a period of 30 months. Independent of glucose control, insulin resistance, adiponectin levels, and LAR are strong markers for liver steatosis in overweight or obese subjects.

## Abbreviations

CAP: Controlled attenuation parameter
E: Young’s modulus
FLI: Fatty liver index
IGT: Impaired glucose tolerance
IR: Insulin resistance
LAR: Leptin/adiponectin ratio
NAFLD: Nonalcoholic fatty liver disease
NFS: NAFLD fibrosis score
NGT: Normal glucose tolerance
T2DM: Type 2 diabetes mellitus
